# MTL-DoHTA: Multi-Task Learning-Based DNS over HTTPS Traffic Analysis for Enhanced Network Security

**DOI:** 10.3390/s25040993

**Published:** 2025-02-07

**Authors:** Woong Kyo Jung, Byung Il Kwak

**Affiliations:** Division of Software, Hallym University, Chuncheon 24252, Republic of Korea; m24053@hallym.ac.kr

**Keywords:** DNS over HTTPS, DNS covert channel, multi-task learning, deep learning

## Abstract

The adoption of DNS over HTTPS (DoH) has significantly enhanced user privacy and security by encrypting DNS queries. However, it also presents new challenges for detecting malicious activities, such as DNS tunneling, within encrypted traffic. In this study, we propose MTL-DoHTA, a multi-task learning-based framework designed to analyze DoH traffic and classify it into three tasks: (1) DoH vs. non-DoH traffic, (2) benign vs. malicious DoH traffic, and (3) the identification of DNS tunneling tools (e.g., dns2tcp, dnscat2, iodine). Leveraging statistical features derived from network traffic and a 2D-CNN architecture enhanced with GradNorm and attention mechanisms, MTL-DoHTA achieves a macro-averaging F1-score of 0.9905 on the CIRA-CIC-DoHBrw-2020 dataset. Furthermore, the model effectively handles class imbalance and mitigates overfitting using downsampling techniques while maintaining high classification performance. The proposed framework can serve as a reliable tool for monitoring and securing sensor-based network systems against sophisticated threats, while also demonstrating its potential to enhance multi-tasking capabilities in resource-constrained sensor environments.

## 1. Introduction

The increasing importance of internet security and privacy has made the Domain Name System (DNS) a critical but vulnerable target for cyberattacks. As the backbone of the Internet, DNS translates human-readable hostnames into machine-readable IP addresses, enabling seamless and efficient communication between users and websites. However, the traditional DNS design transmits data in plaintext, making it susceptible to threats like eavesdropping, data manipulation, and Man-in-the-Middle (MitM) attacks [1]. Attackers have exploited these vulnerabilities to intercept sensitive information or manipulate DNS responses for malicious purposes, such as redirecting users to phishing websites or delivering malware. Recognizing the need to secure DNS communications, the Internet Engineering Task Force (IETF) introduced DNS over HTTPS (DoH) in 2018 [2]. This standardized protocol encrypts DNS queries using HTTPS, ensuring that communication between users and DNS resolvers remains confidential and protected against interception, thus significantly enhancing user privacy and security [3].

Despite its benefits, DoH poses new challenges for network security. By encrypting DNS traffic and embedding it within HTTPS, DoH obscures the visibility of DNS queries, making them indistinguishable from other web traffic [4]. This encryption prevents traditional packet inspection techniques from identifying DNS-related activities, complicating detecting and mitigating malicious behaviors. Attackers have exploited this characteristic to perform DNS tunneling, a method that uses DNS queries to covertly transfer data or communicate with command and control (C2) servers [4,5]. Such misuse has been observed in high-profile cases, including Godlua malware and Oilrig (APT34), where DoH was used to exfiltrate data and maintain C2 channels [6,7]. These incidents highlight a critical gap in existing DNS traffic analysis methods as they struggle to address the complexities introduced by encrypted DNS traffic.

In light of these challenges, research has shifted towards developing more robust detection mechanisms. Machine learning (ML) and deep learning (DL) approaches are increasingly employed to analyze encrypted DNS traffic. As noted by Jehad Ali et al. [8], advanced ML algorithms, such as anomaly detection and behavior analysis, have shown promise in identifying deviations from normal patterns within encrypted communications. Such approaches enable the detection of sophisticated threats while maintaining privacy safeguards. Ali et al. emphasize the importance of integrating AI-driven security frameworks, particularly in environments with significant IoT and network interconnectivity, such as smart cities. Their work highlights the role of adaptive AI systems in continuously learning and evolving to address emerging cyber threats.

Furthermore, while meta-learning frameworks have demonstrated efficacy in detecting intrusions and abnormal encrypted network traffic in IoT environments [9], their applicability to DoH remains underexplored. As DoH evolves, methodologies that enhance adaptability to new DNS tunneling technologies and attack vectors need to be incorporated. This adaptability can be achieved through multi-task learning frameworks that allow models to generalize across diverse scenarios without compromising detection accuracy or scalability.

Developing advanced detection mechanisms requires a multifaceted approach that combines machine learning algorithms with behavioral analysis to identify anomalies in DNS traffic patterns while maintaining strict privacy safeguards. These mechanisms must enhance model performance and preserve user privacy, a cornerstone of encrypted DNS traffic such as DoH. Furthermore, security solutions need to remain adaptable and effective even in diverse and dynamic network environments, providing a critical layer of resilience against the evolving landscape of cyber threats.

Motivated by these challenges, we propose multi-task learning (MTL)-based traffic classification model to address the complexities of DoH traffic detection and malicious activity identification. The MTL model is designed to tackle three interconnected tasks: (1) classifying network traffic into DoH and non-DoH categories, (2) distinguishing between benign and malicious DoH traffic, and (3) conducting a multi-class classification to identify specific DNS tunneling tools used in malicious activities. Leveraging time-series classification techniques, our MTL model offers comprehensive insight into network traffic, enabling rapid and accurate detection across all tasks. By achieving the high performance of single-task models while simultaneously addressing multiple objectives, this MTL-based approach enhances network security by effectively identifying malicious behaviors and specific DNS tunneling tools while preserving the privacy benefits of DoH.

The research contributions of the multi-task learning-based traffic classification model proposed in this study are as follows:

1. It introduces a novel framework that integrates both classification tasks, enabling more efficient learning and improved accuracy in detecting threats within encrypted traffic.
2. The model leverages shared representations across tasks, which not only reduces the computational burden but also enhances generalization capabilities by learning from diverse data patterns present in both benign and malicious traffic.
3. The proposed methodology has been confirmed to achieve high accuracy and superior performance in both learning and testing. This represents a significant contribution to the research, demonstrating the effectiveness of multi-task processing.
4. The framework employs downsampling techniques to address class imbalance in the dataset, ensuring that the model maintains high performance across underrepresented classes. This approach not only improves classification accuracy but also reduces the risk of overfitting, especially in multi-class tasks such as DNS tunneling tool identification.

The rest of this paper is organized as follows. Section 2 reviews the related studies. Section 3 presents our multi-task learning method based on DoH traffic analysis and classification. Section 4 describes the experimental results and evaluates our proposed approach. Finally, Section 5 provides limitations and the concluding remarks.

## 2. Related Work

To comprehensively understand the research themes, characteristics, and limitations of existing studies related to DoH traffic analysis, we added a comparative analysis table that examines whether Tasks 1, 2, and 3 were addressed in prior research, particularly emphasizing the usability of DoH (See Table 1).

The adoption of DoH has significantly enhanced DNS security through encryption, yet it also introduces new challenges for detecting malicious activities such as DNS tunneling. By obfuscating traffic within HTTPS, DoH complicates the ability of existing detection technologies to differentiate DNS requests from standard web traffic, allowing malicious entities to covertly transmit data or obscure communication with C2 servers [1]. DNS tunneling is a technique used to hide malicious data within normal DNS queries. This method allows attackers to bypass firewalls and security measures, which can result in unauthorized data being extracted and malware being activated remotely. Instances of such exploitation underscore the inadequacies of conventional static rule-based detection systems in scrutinizing DoH traffic and accentuate the necessity for more advanced detection methodologies [4].

Recent studies have explored machine learning (ML) techniques to detect malicious DoH traffic, with particular emphasis on time-series analysis. Singh et al. [11] demonstrated that integrating ML algorithms with temporal attributes improves the detection of DNS tunneling activities. Ensemble learning methods such as Gradient Boosting and Random Forest have shown high precision in classifying DoH traffic based on packet dimensions, transmission velocity, and session length [13]. Moreover, some studies have incorporated feature extraction techniques using machine learning and PCAP-based novel features to enhance model performance and improve malicious DoH detection [15].

Building on these techniques, MontazeriShatoori et al. [12] proposed a DoH detection approach together with the CIRA-CICDoHBrw-2020 dataset [17], which contained pre-extracted flow statistics. Their experiments compared multiple ML algorithms (Random Forest, Decision Tree, SVM, Naive Bayes), 2D-CNN, and LSTM; Random Forest achieved a notably high F1-score using 28 features. Furthermore, other research efforts leveraged deep learning architectures. In particular, Singh et al. [11] explored LSTM (Long Short-Term Memory) networks, demonstrating how temporal analysis can help to uncover malicious patterns in DoH traffic.

To further enhance the interpretability of DoH detection models, researchers have integrated visualization techniques. Mohammad et al. [10] performed visualization work on the CIRA-CIC-DoHBrw-2020 dataset (also referred to as CIC-DoHBrw-2020) using Eigen Centrality (EC) in graph/network theory, Principal Component Analysis (PCA), and a Gaussian Mixture Model (GMM). These methods analyzed specific clusters in the data to identify potential anomalies. Similarly, Zebin et al. [16] focused on classifying benign versus malicious DoH using a machine learning–based Random Forest algorithm, augmenting interpretability through Shapley additive explanations (SHAP) and the visualization of packet data. Furthermore, Jerabek et al. [18] performed a comparative analysis on both the CIC-DoHBrw-2020 dataset and a real-world dataset [19], examining the transferability, usability, and longevity of previously published malicious DoH detection machine learning models across these different data sources.

Stalder [15] proposed a three-layered framework to address three distinct classification tasks: DoH vs. non-DoH, Benign vs. Malicious DoH, and DNS tunneling tool classification. This framework integrates ML algorithms tailored for each classification task and employs feature importance analysis during preprocessing to enhance detection accuracy. However, the research lacked results for Task 3 (DNS tunneling tool classification), leaving this aspect unexplored.

Although previous research has contributed to DoH detection, recent studies indicate that existing datasets and models remain insufficient in addressing evolving threats comprehensively. The recent datasets require broader attack vector coverage and improved malicious behavior representations. Moreover, many ML/DL-based models must address task scalability, ensuring that existing models can be reused or extended when new malicious DNS tunneling tools emerge. In response to these gaps, this paper proposes a multi-task learning–based traffic analysis and classification approach which aims to enhance task scalability and adaptability in the face of evolving threats.

## 3. Methodology

**Figure 1:**
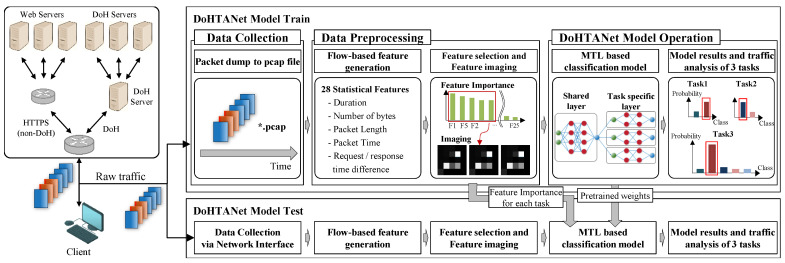
Overview of MTL-DoHTA framework. The * in ‘.pcap’ represents all file names.

In this section, we show the proposed method concerning multi-task learning-based DoH traffic analysis and classification (MTL-DoHTA). Figure 1 shows the overview of our MTL-DoHTA, which integrates various machine learning techniques to simultaneously analyze and classify DoH traffic patterns. By leveraging shared representations across multiple tasks, this approach not only improves the accuracy of detection but also enhances the model’s ability to generalize across different types of network behaviors. The overview is composed three stages: first, the data preprocessing phase, where raw DoH traffic is cleaned and transformed into a suitable format for analysis; second, the feature extraction stage, which identifies key characteristics of the traffic that are critical for effective classification; and third, the model training phase, where machine learning algorithms are employed to learn from the extracted features and optimize performance across tasks.

### 3.1. Data Collection by Traffic Flow Unit

In this subsection, to integrate network traffic into the MTL algorithm, the raw traffic is transformed into a flow-based representation that encapsulates the essential attributes of individual data packets. This representation not only facilitates efficient processing but also enhances the model’s ability to identify patterns and detect anomalies within the traffic, ultimately improving classification accuracy. Once the flow-based features are generated, they undergo a feature extraction process to select only the most critical features. These refined features are then utilized as inputs to the MTL algorithm, ensuring optimal performance in the classification tasks.

### 3.2. Feature Selection with Feature Importance

We extracted a total of 29 features from network traffic using the ‘DoHlyzer’ tool [12], generating flow-based statistical features. The complete list of these features is provided in Table 2. These features are categorized into attributes such as duration, number of bytes, packet length, packet time, and request/response time difference. To ensure model efficiency and lightweight processing, we selected 25 features with the highest feature importance as inputs for the model. This feature selection process is conducted only during the training phase and is not repeated during validation or testing. Instead, the features selected during training are directly used in the validation and testing phases. By focusing on the most relevant information, the selected features enhance the model’s performance and ensure better generalization on unseen data.

**Figure 2:**
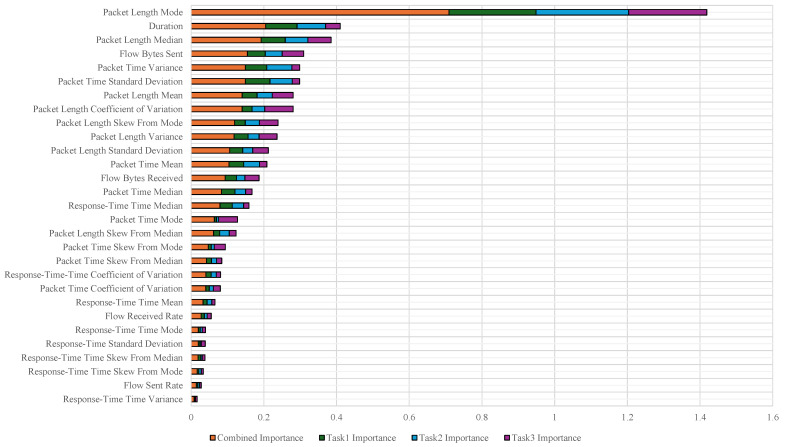
Feature importance via Random Forest algorithm. X-axis is a feature importance value, and Y-axis is a statistical feature name.

To identify the top 25 features out of the initial 29, we applied the Random Forest [20] algorithm as a single-task learning approach to each of the tasks (Task 1, Task 2, and Task 3). Based on the feature importance scores obtained for each task, we identified the top 25 features that were commonly ranked highly across all tasks (see Figure 2). These features, representing the most significant attributes overall, were then used as inputs to the 2D-CNN and attention-based models in this study.

**Figure 3:**
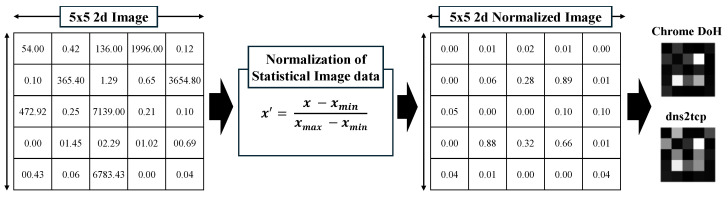
Statistical features to image.

The 25 selected features undergo a MinMax normalization process, where their values are scaled between 0 and 1 according to Equation (1), as shown in Figure 3. After normalization, the features are arranged sequentially into a 5 × 5 vector, resulting in an image-like representation.

(1) $$x^{\prime }=\frac{x-x_{\min}}{x_{\max}-x_{\min}}$$

### 3.3. MTL-DoHTA Model

**Figure 4:**
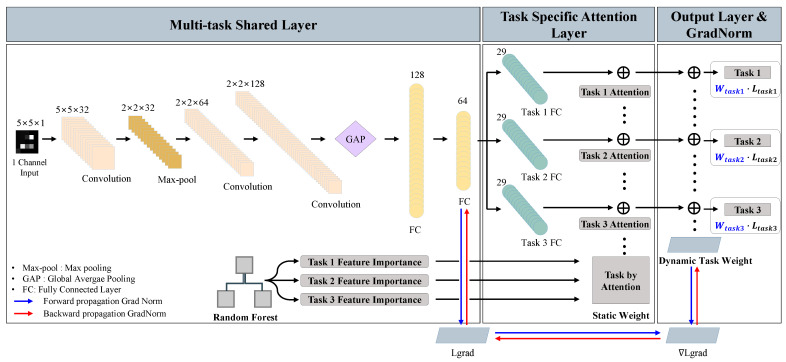
Structure of MTL-DoHTA.

To enable the simultaneous processing of multiple tasks, we designed the architecture of a MTL algorithm, as illustrated in Figure 4. The proposed MTL algorithm consists of three main components: 1. A shared network architecture in the neural network structure; 2. a task-specific attention architecture for each task; 3. an output layer that computes the outputs and dynamically updates task weights using GradNorm [21].

The proposed architecture leverages a shared network to learn common features across tasks, thereby enhancing the model’s generalization capability. Increasing the width of the shared network can further improve its ability to generalize; however, overly generalized shared features may lack robustness in capturing task-specific characteristics. To address this, the network incorporates dedicated task-specific layers, structured as a multi-task shared layer, a task-specific attention layer, and an output layer with GradNorm-based weight updates. The task-specific attention layer utilizes precomputed and fixed attention weights to assign feature importance for each task. These static attention weights guide the model in updating network parameters effectively, ensuring that each layer focuses on task-specific information based on pre-extracted features. Meanwhile, the output layer employs task-specific loss functions to compute final losses and predictions. In addition, the gradient norms of the final dense layer in the shared network are computed to compare the gradient norms across tasks. This enables dynamic updates of task weights using GradNorm, ensuring balanced learning among the tasks. The components and their detailed functionalities are described in the subsequent sections.

#### 3.3.1. Multi-Task Shared Layer

The shared layer is responsible for extracting common features from the input data in the early stages of the network. In this study, the convolutional networks in the shared layer employ 3 × 3 filters and are structured to enhance feature extraction efficiency. The shared layer consists of three convolutional layers, a max-pooling layer, and two fully connected layers, with ReLU activation functions incorporated into the 2D-CNN structure to introduce non-linearity and enable the network to learn diverse features.

The input data, represented as a 2D image, sequentially pass through four shared layers before entering the task-specific layers. The first shared layer expands the 5 × 5 × 1 input into 32 feature maps and compresses it using a max-pooling layer, reducing the spatial dimensions to 2 × 2. The second and third shared layers further expand the number of feature maps to 64 and 128, respectively, progressively capturing more abstract representations. Following this, a global average pooling (GAP) layer computes the average values of the output feature maps. It transforms them into a 128-dimensional dense vector, a predefined size independent of the feature map dimensions. The flattened vector is then processed through the fully connected layers, where a 64-unit shared fully connected layer compresses the representation before passing it into the task-specific attention layer.

#### 3.3.2. Task Specific Attention Layer

The task-specific attention layer consists of separate, fully connected layers, each with 29 units corresponding to the number of features used in this study. Each task-specific layer functions as an attention mechanism, where precomputed feature importance values are statically multiplied by fixed attention weights throughout the training process. This allows each task to focus on the most relevant features while preserving the structural integrity of the shared representation.

After passing through the attention layer, Task 1 and Task 2 employ the BCEWithLogits loss function for independent binary classification, generating probability values for classification decisions. In contrast, Task 3 utilizes the cross-entropy loss function to classify inputs into five categories, effectively capturing multi-class relationships.

#### 3.3.3. Static Attention Mechanism Based on Feature Importance

In this study, the feature importance values for each task are precomputed using the Random Forest algorithm and utilized as prior knowledge during training. Instead of dynamically learning the attention weights, the model leverages task-specific feature importance to guide specialized training for each task.

The reasons for not dynamically learning attention weights are as follows:

- The robust weights learned through the shared layer provide generalized representation power across all tasks, while the additional attention layer further emphasizes this generalized representation.
- Dynamically learning attention weights can significantly increase computational costs, especially when combined with the computations required for the shared layer. This consideration makes static attention weights a more efficient choice.
- By explicitly reflecting the important features, the model ensures task-specific alignment, allowing each task to focus on its most relevant features without additional complexity.

#### 3.3.4. Attention Mechanism

To enhance the learning effectiveness of our MTL-DoHTA model, the attention mechanism enables the model to focus on the most relevant parts of the input data, thereby improving its performance [22]. This approach assigns attention weights to the encoder’s hidden states, emphasizing the importance of each input token during decoding.

First, we compute a weighted sum of the encoder’s hidden states, guided by these attention weights. Let this weighted sum be denoted by *z*, as defined in Equation (2):

(2) $$z=W_{attention}\cdot x$$

Here, *x* represents the input network weights, which are learned through backpropagation. Next, to ensure that the resulting attention weights sum to 1, we apply a softmax function to *z* (Equation (3)):

(3) $$\mathrm{attention}\_{\mathrm{scores}}_{i}=\frac{\exp(z_{i})}{\sum_{j}\exp\left(z_{j}\right)}\;\mathrm{for}\;i\in \left[1,n\right]$$

producing a probability distribution that indicates how much attention is allocated to each token. Finally, we multiply the encoder’s hidden states by these normalized attention scores in an element-wise manner to obtain the context vector $X_{attended}$, as shown in Equation (4):

(4) $$x_{attended}=x⊙\mathrm{attention}\_\mathrm{scores}$$

This context vector highlights the most important features of the input data for predicting the current output, enabling the MTL-DoHTA model to adaptively focus on different tokens at each step.

#### 3.3.5. Output Layer and GradNorm for Dynamic Task Weighting

After the fully connected layer for each task, the loss values are calculated using the respective loss functions. During the backward pass, the gradient norms of the fully connected network weights in the last shared layer (64 units) are computed. These gradient norms are normalized to a common scale and multiplied by the relative inverse training rate of each task. As a result, the task weights are dynamically adjusted during backpropagation based on these common-scale gradient norms. In multi-task learning, the final loss function is typically the sum of the loss values for each task. To account for differing learning speeds among tasks, task weights $w_{i}$ are introduced to regulate each task’s contribution to the total loss. Equation (5) shows this weighted multi-task loss function, where *i* indexes each task:

(5) $$L_{MTL}=\sum_{i}w_{i}\cdot L_{i}$$

GradNorm is a normalization technique designed to balance the loss values across tasks by directly tuning $w_{i}$ based on the gradient magnitudes of the shared layer. Unlike grid search, GradNorm uses a single hyperparameter α to adjust task weights dynamically, allowing tasks with slower learning rates to catch up and train at a pace similar to other tasks. The GradNorm update rule for each task weight $w_{i}$ is shown in Equation (6):

(6) $$w_{i}\left(t+1\right)=w_{i}\left(t\right)+\alpha \cdot \left(G_{i}\left(t\right)-\bar{G}\left(t\right)\right)$$

Here, $G_{i}\left(t\right)$ indicates the L2 norm of the gradients associated with task *i*. Although some approaches [22] suggest using the entire shared layer for this computation, we focus on the gradients in the last shared layer only for efficiency. Equation (7) defines $G_{i}\left(t\right)$:

(7) $$G_{i}\left(t\right)={∥\nabla_{W}L_{i}∥}_{2}$$

The common scale $\bar{G}\left(t\right)$ is then computed as the average of these gradient norms across all *T* tasks (Equation (8)):

(8) $$\bar{G}\left(t\right)=\frac{1}{T}\sum_{i=1}^{T}G_{i}\left(t\right)$$

In addition to gradient norms, GradNorm also calculates a loss ratio, $L_{i}\left(t\right)$, which represents how much the loss for task *i* has changed relative to its initial value. Equation (9) shows how $L_{i}\left(t\right)$ is derived:

(9) $$L_{i}\left(t\right)=\frac{L_{i}\left(t\right)}{L_{i}\left(0\right)}$$

A smaller $L_{i}\left(t\right)$ implies faster convergence (lower loss over time), whereas a larger $L_{i}\left(t\right)$ indicates slower learning. GradNorm then uses the relative inverse training rate, $r_{i}\left(t\right)$, to measure how a task’s progress compares to the overall average loss, as shown in Equation (10):

(10) $$r_{i}\left(t\right)=\frac{L_{i}\left(t\right)}{\bar{L}\left(t\right)}$$

Here, $\bar{L}\left(t\right)$ is the average loss across all tasks. If $r_{i}\left(t\right)>1$, it suggests that the task *i* is learning more slowly than average and therefore requires more attention (i.e., a higher weight). GradNorm leverages these metrics to adjust $w_{i}$ so that all tasks can maintain a balanced learning pace. In addition to adjusting the task weights, GradNorm also updates the gradient norm for each task to reflect this inverse training rate. As shown in Equation (11), the gradient norm $G_{i}\left(t\right)$ is shifted closer to the common scale $\bar{G}\left(t\right)$ based on α and $r_{i}\left(t\right)$:

(11) $$G_{i}\left(t+1\right)=G_{i}\left(t\right)+\alpha \cdot \left(r_{i}\left(t\right)\cdot \bar{G}\left(t\right)-G_{i}\left(t\right)\right)$$

Here, α is a hyperparameter that controls how strongly the gradient update prioritizes tasks with higher losses. Tasks displaying slower learning rates ($r_{i}\left(t\right)>1$) thus receive proportionally larger adjustments in their gradient norms. Finally, GradNorm defines a gradient loss $L_{grad}$ that quantifies the discrepancy between the individual gradient magnitudes $G_{i}\left(t\right)$ and the rescaled common scale $\bar{G}\left(t\right)$. Equation (12) shows how $L_{grad}$ is computed:

(12) $$L_{grad}=\sum_{i}\left|G_{i}\left(t\right)-\bar{G}\left(t\right)\right|$$

By minimizing $L_{grad}$, the method encourages each task’s gradient norm to remain close to the overall average, ensuring that all tasks progress at a similar pace. The task weights are normalized at each time step so that their sum equals *T*, the total number of tasks. The overall process of the MTL-DoHTA model is illustrated in Algorithms 1 and 2, detailing both the forward pass and backward propagation steps.
**Algorithm 1** DoHTA Multi-task Learning Forward Pass  1:**Input:** $X\in R^{B\times 5\times 5\times 1}$
  2:**Output:** $\left\{y_{pred1},y_{pred2},y_{pred3}\right\}$
  3:Permute *X* to (B,1,5,5)▹ Reorganize input tensor  4:$X_{conv1}\leftarrow \mathrm{ReLU}\left({\mathrm{Conv}2D}_{1\to 32}\left(X\right)\right)$▹ Output channels = 32  5:$X_{pool}\leftarrow \mathrm{MaxPool}\left(X_{conv1}\right)$▹ Downsample to 2 × 2  6:$X_{conv2}\leftarrow \mathrm{ReLU}\left({\mathrm{Conv}2D}_{32\to 64}\left(X_{pool}\right)\right)$▹ Output channels = 64  7:$X_{conv3}\leftarrow \mathrm{ReLU}\left({\mathrm{Conv}2D}_{64\to 128}\left(X_{conv2}\right)\right)$▹ Output channels = 128  8:$X_{gap}\leftarrow \mathrm{Global}\;\mathrm{Average}\;\mathrm{Pooling}\left(X_{conv3}\right)$▹ Global Average Pooling  9:$X_{fc1}\leftarrow \mathrm{Dropout}\left(\mathrm{ReLU}\left(X_{gap}\right)\right)$
10:$X_{shared}\leftarrow \mathrm{Dropout}\left(\mathrm{ReLU}\left(X_{fc1}\right)\right)$▹ Shared output for task-specific heads11:**for** i∈{1,2,3} **do**
12:    $\alpha^{\left(i\right)}\leftarrow \mathrm{Softmax}\left(X_{shared}A^{\left(i\right)}\right)$▹ Static feature importance $A^{\left(i\right)}$13:    $X_{task}^{\left(i\right)}\leftarrow X_{shared}⊙\alpha^{\left(i\right)}$▹ Attention applied for task *i*14:**end for**
15:$y_{pred1}\leftarrow \mathrm{BCEWithLogits}\left(X_{task1}\right)$
16:$y_{pred2}\leftarrow \mathrm{BCEWithLogits}\left(X_{task2}\right)$
17:$y_{pred3}\leftarrow \mathrm{CrossEntropy}\left(X_{task3}\right)$**    return** $\left\{y_{pred1},y_{pred2},y_{pred3}\right\}$

**Algorithm 2** DoHTA GradNorm Backward Propagation  1:**Input:**     Task losses $L_{task1},L_{task2},L_{task3}$     Shared output $X_{shared}$     Task weights $w_{task1},w_{task2},w_{task3}$  2:**Output:**     Total loss $L_{total}$     Updated task weights $w_{task1},w_{task2},w_{task3}$  3:**Step 1: Compute Total Loss**  4:$L_{total}\leftarrow w_{task1}L_{task1}+w_{task2}L_{task2}+w_{task3}L_{task3}$  5:**Step 2: Backpropagate Gradients for Shared Output**  6:Compute gradients of total loss with respect to $X_{shared}$:  7:$X_{shared}\leftarrow \nabla_{X_{shared}}\left(w_{task1}L_{task1}+w_{task2}L_{task2}+w_{task3}L_{task3}\right)$  8:**Step 3: GradNorm Application**  9:Compute updated task weights using GradNorm function:10:$w_{task1},w_{task2},w_{task3}\leftarrow \mathrm{GradNorm}\left(L_{task1},L_{task2},L_{task3},X_{shared}\right)$11:**Step 4: Return Values**12:**return** Total loss $L_{total}$ and updated task weights $w_{task1},w_{task2},w_{task3}$

## 4. Experimental Evaluation

### 4.1. Dataset and Performance Metrics

The CIC-DoHBrw-2020 dataset, developed by the Canadian Institute for Cybersecurity Research, provides valuable insights, detailed in Table 3 [12]. It includes DoH traffic generated using Google Chrome, Mozilla Firefox, and three DNS covert channel tools: iodine, dnscat2, and dns2tcp. This traffic interacts with four DoH servers, namely AdGuard, Cloudflare, Google DNS, and Quad9, to capture diverse behaviors. The dataset is organized into three categories: non-DoH (regular HTTPS traffic), benign-DoH (normal DoH traffic), and malicious-DoH (DoH-encrypted DNS covert channels). While non-DoH and benign-DoH traffic are created by accessing Alexa’s top 10,000 domains, malicious DoH traffic is generated by covert channel tools using TLS-encrypted HTTPS requests to specific DoH servers. To train our model, we divided the CIC-DoHBrw-2020 dataset 8:2 in the experiment. The total train data comprised 927,419 (80%), and the test data comprised 231,822 (20%). The experiment was operated on a system with Windows 11 OS, an Intel(R) Core i9-14900KF processor, and a Geforce RTX 4090 GPU, using Python 3.9 (see Table 4).

### 4.2. Hyperparameter Settings

To calculate the importance for each single task, the Random Forest algorithm was used with default hyperparameter settings. Specifically, the number of decision trees was set to 100, the maximum tree depth was “unlimited”, and Gini impurity was applied. For the MTL DNN algorithm, the following hyperparameters were used: a dropout rate of 0.3, a batch size of 32, and an Adam optimizer. The scaling factor α for GradNorm, which balances tasks, was set to 1.9. GradNorm weights were updated every 10 batch sequences to naturally integrate with the mini-batch gradient descent method. Training was conducted for 50 epochs with a learning rate of 0.001, and the final evaluation was based on the model achieving the highest total F1-score during these epochs. Additionally, the Optuna library [23] was used to optimize the hyperparameters, including the learning rate, batch size, and dropout rate. The search ranges were as follows:

- Learning rate: [1 × 10^−4^, 1 × 10^−2^];
- Batch size: [16, 32, 64];
- Dropout rate: [0.1, 0.5].

To obtain the optimal hyperparameters, we set another dataset that was a 50% downsampled version of the previous train dataset. To further analyze the model’s performance, the structural parameters of the MTL-DoHTA model, which play a critical role in processing these datasets, are detailed in Table 5.

### 4.3. Performance Evaluation

To validate the performance of our proposed model, we conducted evaluations from three perspectives:

1. We assessed task-specific performance based on changes in the layer width of the model’s shared structure.
2. We evaluated the performance improvements resulting from applying GradNorm and the attention mechanism to the baseline 2D-CNN architecture.
3. We examined the model’s performance when using downsampling to address class imbalance and prevent overfitting caused by data redundancy.

In the first evaluation, we analyzed the F1-score for each task based on changes in the convolutional structure of the shared layer (see Table 6). As shown in Table 6, increasing the width of the shared layer consistently improved performance across all tasks.

For the second evaluation, we measured the performance improvements from applying GradNorm and the attention mechanism to the baseline 2D-CNN architecture. The results show that adding GradNorm to the 2D-CNN significantly improved the model’s performance. Furthermore, applying both GradNorm and the attention mechanism yielded the highest performance. While the F1-score for Task 2 in the baseline 2D-CNN (32-64-128) was comparable to that of the MTL-DoHTA model, the F1-scores for Task 1 and Task 3 were noticeably higher with MTL-DoHTA.

In the third evaluation, we explored the performance of both the baseline model and MTL-DoHTA with varying downsampling rates (see Table 7). Downsampling offers advantages such as addressing class imbalance, reducing computational resources, and preventing overfitting. However, it can also lead to performance degradation due to insufficient training data. Despite this, as shown in Table 7, the MTL-DoHTA model maintained robust performance even with a downsampling percentage of 50%. Specifically, addressing class imbalance through downsampling resulted in only a minor decrease in F1-score, with an average difference of just 0.003 compared to using the full dataset. This demonstrates that MTL-DoHTA effectively mitigates the impact of downsampling.

Table 8 presents the performance comparison results for different attention weight selection strategies applied in the MTL-DoHTA model. As shown in the Table 8, the highest macro-averaging F1-score of 0.9905 was achieved when applying the 2D-CNN + Grad-Norm + Attention mechanism with static attention weights based on feature importance. Furthermore, it can be observed that increasing the width of the shared layer consistently improved the model’s performance.

The proposed model in this study was evaluated for prediction time performance. The dataset used for training and prediction did not undergo downsampling, and the full dataset was used for evaluation. From the full dataset, 20% (231,822 flows) was selected, and the prediction process was repeated 1000 times to calculate the average prediction time. The average prediction time was recorded as 0.021379 s.

### 4.4. Comparison with Other Methods

Along with our proposed MTL-DoHTA model with 2D-CNN, GradNorm, and Static attention weight (feature importance), we have also compared it with other study methods. As shown in Table 9, we confirmed that our proposed MTL-DoHTA model has subtle differences from different studies in terms of performance on Task 1 and Task 2. However, in other studies, the F1-score of Task 3, which was not focused on, was high at 0.9837. Since the study by Liu et al. [5] used an algorithm that applied few shots, it is somewhat limited to directly comparing the performance with this paper’s algorithm.

In addition, we confirmed the differences between our study and previous studies through a comparative analysis of model complexity, scalability, and the number of model parameters (see Table 10). MontazeriShatoori et al. [12] used approximately 37,000 parameters, showing that the model size is relatively smaller than the algorithms of other studies. Our proposed model uses 105,546 parameters, and the total model size is approximately 450KB, a small resource requirement that allows the model to be sufficiently executed on embedded devices. Although many methodologies and algorithms have been proposed in previous studies, they were not shown in Table 10 because the model size of machine learning algorithms varies depending on the learning criteria of the records. When looking at the complexity of the model, excluding the number of parameters, deep learning-based methods have a part where the complexity of the model changes depending on the layer width and depth settings. Accordingly, the model proposed in this study is relatively different. Compared to deep learning algorithms, it has low complexity, and in the case of deep learning algorithms with simple structures, the model complexity can be expressed as middle. In addition, since it is more free to update the model output layer in a new environment or when a new task appears, it has been shown to have high scalability in this study. However, general machine learning-based algorithms are performed as a single task even when performing multi-class classification, and there is a cumbersome part in that a new algorithm must be re-learned for other tasks, so in general, the model appears to have low scalability.

### 4.5. Performance Comparison in Two Datasets

To evaluate the generalization performance of the proposed MTL-DoHTA model, experiments were conducted on two datasets: the DNS Over HTTPS network traffic [25] and CIRA-CIC-DoHBrw-2020 and DoH-Tunnel-Traffic-HKD combined dataset [26,27]. The first dataset, the IEEE Dataport Dataset, will be referred to as Dataset 1 for simplicity. Similarly, the second dataset, CIRA-CIC-DoHBrw-2020 and DoH-Tunnel-Traffic-HKD combined dataset will be referred to as Dataset 2 throughout the remainder of this section.

Dataset 1 was used to assess the model’s performance across various tasks, while the DoH Tunnel Traffic HKD dataset introduced a new DNS tunneling technique to evaluate the model’s adaptability to novel threats. For Dataset 1, we pre-trained the MTL-DoHTA model and fine-tuned it over 100 epochs to ensure sufficient training. The model achieved an average F1-score of 0.9863, with Task 1 scoring 0.9841 and Task 3 scoring 0.9907. This dataset, which supports flow-based processing with Pcap and includes diverse DNS resolvers, allowed comprehensive evaluations for Task 1 and Task 3. However, due to the absence of malicious DoH tunneling tools, Task 2 evaluations were limited in scope and feasibility. Dataset 2, which augments the CIC-DoHBRW-2020 dataset by including a new type of malicious DoH tunneling tool, was used to assess the model’s performance specifically for Task 3. Since Task 1 and Task 2 of Dataset 2 align with the existing CIC-DoHBRW-2020 dataset, fine-tuning was limited to 10 epochs to preserve computational efficiency. The model achieved an impressive F1-score of 0.9996 for Task 3, demonstrating its effectiveness in identifying new tunneling techniques.

## 5. Conclusions

The proposed MTL-DoHTA framework effectively classifies DNS over HTTPS (DoH) traffic across three tasks: (1) differentiating DoH vs. non-DoH traffic, (2) classifying benign vs. malicious DoH traffic, and (3) identifying DNS tunneling tools such as dns2tcp, dnscat2, and iodine. By leveraging statistical features and a simple 2D-CNN architecture, MTL-DoHTA achieves a macro-averaging F1-score of 0.9905 on the CIC-DoHBrw-2020 dataset, outperforming GradNorm and static attention-based methods and thus demonstrating robustness and adaptability.

Despite these achievements, applying MTL-DoHTA in real-time environments presents challenges due to the reliance on pre-extracted features and the computational complexity of the network, potentially hindering deployment in latency-sensitive scenarios. Moreover, retraining is required to adapt to novel or evolving DNS tunneling tools. To address these limitations, future work will prioritize lightweight model optimization and explore continual learning approaches to enhance real-time detection, adaptability, and scalability. Our next experimental phase will also include testing with a real-nature dataset and additional tasks to further validate the model’s performance under diverse conditions.

The effectiveness of the 2D-CNN-based MTL-DoHTA model was validated through comparisons with baseline models. However, the model’s complex structure imposes limitations on real-time performance, which remains an area for improvement. Future research will focus on enhancing both the accuracy and real-time efficiency of the proposed approach by investigating more compact network designs and incremental training strategies.

## Figures and Tables

**Table 1:** Literature review via characteristics and limitations related to DoH.

Study	Research Themes	Characteristics	Limitations	Task 1	Task 2	Task 3
[1]	Investigating preliminary identification methodologies for the recognition of DoH utilization.	Acknowledge the significance of early detection of DoH traffic and elucidate fundamental detection methodologies.	Preliminary investigations exhibit inadequate detection precision, and there exists an absence of systematic classification and comprehensive examination of malevolent traffic.	V	X	X
[10]	Centers on advanced visualization (Eigen Centrality, PCA, GMM) and dataset exploration for IDS enhancements in DoH-based cyber threats.	Demonstrates the importance of Layer 3 data and realistic threat simulation to inform the development of more effective IDS models.	Dataset imbalances, inconsistent classification performance across varied methods, and limitations of task scalability	X	V	X
[4]	Analyze how encrypted DNS queries are used in botnets and other malicious activities	Presenting techniques and countermeasures for botnet activities and DNS tunneling exploiting DoH	Discuss misuse cases rather than detection techniques	V	V	X
[5]	Proposal of a DNS tunneling detection framework that integrates model-agnostic meta-learning and Convolutional Neural Networks.	Facilitates elevated precision via swift adjustment within constrained data contexts.	The emphasis is placed on particular DNS tunneling instruments, resulting in the generalized detection paradigm lacking scalability.	V	V	X
[6]	Research on methodologies for identifying nefarious DoH traffic through the application of machine learning algorithms.	Examine a range of machine learning frameworks and propose strategies for enhancing detection precision.	Concentrate on binary classification instead of multi-class classification.	V	V	X
[11]	Identify nefarious behavior within DoH traffic by employing ensemble machine learning methodologies.	Conduct a comparative analysis of the efficacy of various models, documenting elevated detection accuracy alongside minimal rates of false positives.	The emphasis is placed on binary classification as opposed to multi-class classification.	V	V	X
[12]	Proposed temporal series classification framework for the identification of DNS tunneling phenomena occurring within DoH traffic.	Enhance precision and operational efficacy through the recommendation of detection methodologies grounded in time series classification algorithms.	No experiments for Task 3 to identify specific DNS tunneling tools	V	V	X
[13]	Research on methodologies for DNS covert channel detection with Multi-layer perceptron, Multi-Head Attention, and Residual Neural Networks	Feature fusion of session feature and sequence feature	Single tasks in multi-class classification and limitation of model’s scalability	V	V	V
[14]	Focuses on simple recurrent neural network multi-stage classification (Task 1 and Task 2) for malicious DoH detection.	Employs the CIC-DoHBrw-2020 dataset with LSTM/GRU models, emphasizing preprocessing, class imbalance handling, and two-layer classification.	Single tasks in RNNs algorithms (LSTM, GRU, deepRNN, and biLSTM) and limitation of model’s scalability	V	V	X
[15]	Focuses on machine-learning detection of malicious DoH traffic, emphasizing a two-step classification (benign vs. malicious DoH).	Implements a PCAP-based novel feature extraction and ML (e.g., LGBM) to identify malicious DoH activity.	Reduced accuracy across diverse datasets, limited realism in browser settings, and no evaluation of Task 3.	V	V	X
[16]	Proposes an explainable AI framework using a balanced Random Forest to accurately detect and classify malicious DoH traffic.	Leverages the CIC-DoHBrw-2020 dataset, achieves high metrics, and employs SHAP for transparent model decisions.	Lacks of large-scale deployment considerations, and limitations model’s task scalability	V	V	X

**Table 2:** List of statistical traffic features.

Category	Number	Statistical Feature Name
Duration	1	Flow duration
Number of bytes	2	Number of flow bytes sent
	3	Rate of flow bytes sent
	4	Number of flow bytes received
	5	Rate of flow bytes received
Packet length	6	Mean packet length
	7	Median packet length
	8	Mode packet length
	9	Variance of packet length
	10	Standard deviation of packet length
	11	Coefficient of variation of packet length
	12	Skew from median packet length
	13	Skew from mode packet length
Packet time	14	Mean packet time
	15	Median packet time
	16	Mode packet time
	17	Variance of packet time
	18	Standard deviation of packet time
	19	Coefficient of variation of packet time
	20	Skew from median packet time
	21	Skew from mode packet time
Request/responsetime difference	22	Mean request/response time difference
	23	Median request/response time difference
	24	Mode request/response time difference
	25	Variance of request/response time difference
	26	Standard deviation of request/response time difference
	27	Coefficient of variation of request/response time difference
	28	Skew from median request/response time difference
	29	Skew from mode request/response time difference

**Table 3:** CIC-DoHBrw-2020 dataset.

Browsers/Tools	Benign-DoH	Non-DoH	Malicious DoH		
	Google Chrome/Mozilla Firefox	Google Chrome/Mozilla Firefox	iodine	dnscat2	dns2tcp
Number of Flows	19,807	897,493	46,613	35,622	167,515

**Table 4:** Experimental settings.

Category	Experimental Environment
Operating system	Windows 11
Processor	Intel (R) Core™ i9-14900KF
GPU	GeForce RTX 4090
Programming language and version	Python 3.9
Library	Pytorch, scikit-learn

**Table 5:** Structural parameters in MTL-DoHTA.

Structure	Layer	Operation	Input	Output
Shared Layers	Conv2D	2D Convolution (32 filters, 3 kernels, 1 padding)	5×5×1	5×5×32
	ReLU + MaxPool	ReLU Activation + Max Pooling (2 kernels)	5×5×32	2×2×32
	Conv2D	2D Convolution (64 filters, kernel = 3, padding = 1)	2×2×32	2×2×64
	ReLU	ReLU Activation	2×2×64	2×2×64
	Conv2D	2D Convolution (128 filters, kernel = 3, padding = 1)	2×2×64	2×2×128
	ReLU	ReLU Activation	2×2×128	2×2×128
	Global Average Pooling	Pooling over spatial dimensions	2×2×128	128×1
	Fully Connected (fc1)	Linear transformation + ReLU + Dropout	128×1	64×1
	Fully Connected (fc2)	Linear transformation + ReLU + Dropout	64×1	29×1
Task-Specific Attention	Task 1 Attention	Weighted Attention using Task Importance	29×1	29×1
	Task 2 Attention	Weighted Attention using Task Importance	29×1	29×1
	Task 3 Attention	Weighted Attention using Task Importance	29×1	29×1
Task-Specific Heads	Task 1 Head	Linear transformation	29×1	1×1
	Task 2 Head	Linear transformation	29×1	1×1
	Task 3 Head	Linear transformation (Softmax)	29×1	5×1

**Table 6:** Performance of F1-score comparison changing layer structure and function adaptation.

Shared Layer (Layer 1–2–3)	2D-CNN		2D-CNN + GradNorm		MTL-DoHTA (2D-CNN + GradNorm + Attention)	
	Each Task	Average	Each Task	Average	Each Task	Average
16–32–64	Task 1: 0.9785	0.9817	Task 1: 0.9765	0.9775	Task 1: 0.9868	0.9863
	Task 2: 0.9951		Task 2: 0.9922		Task 2: 0.9968	
	Task 3: 0.9715		Task 3: 0.9638		Task 3: 0.9754	
32–32–64	Task 1: 0.9823	0.9852	Task 1: 0.9849	0.9871	Task 1: 0.9834	0.9860
	Task 2: 0.9963		Task 2: 0.9978		Task 2: 0.9969	
	Task 3: 0.9769		Task 3: 0.9785		Task 3: 0.9777	
32–64–128	Task 1: 0.9838	0.9871	Task 1: 0.9864	0.9881	Task 1: 0.9891	0.9905
	Task 2: 0.9988		Task 2: 0.9984		Task 2: 0.9988	
	Task 3: 0.9786		Task 3: 0.9794		Task 3: 0.9837	

**Table 7:** Performance evaluation (F1-score) by downsampling rate. In the 2D-CNN, we choose the shared layer’s width 16–32–64 to set the baseline.

Model	Tasks	Downsampling Percentage				
		10%	20%	30%	40%	50%
2D-CNN	Task 1	0.9874	0.9857	0.9858	0.9860	0.9841
	Task 2	0.9968	0.9975	0.9868	0.9979	0.9946
	Task 3	0.9776	0.9788	0.9740	0.9753	0.9699
MTL-DoHTA (2D-CNN + GradNorm + Attention)	Task 1	0.9853	0.9855	0.9852	0.9865	0.9855
	Task 2	0.9982	0.9977	0.9978	0.9981	0.9983
	Task 3	0.9786	0.9786	0.9763	0.9783	0.9787

**Table 8:** Macro-averaging F1-score of MTL-DoHTA with attention weights (baseline: 2D-CNN + GradNorm).

Shared Layer (Layer 1–2–3)	Baseline	Baseline + Dynamic Attention Weight	Baseline + Static Attention Weight (Average Weight Value)	Baseline + Static Attention Weight (Feature Importance)
16–32–64	0.9775	0.9819	0.9832	0.9863
32–32–64	0.9871	0.9846	0.9866	0.9860
32–64–128	0.9881	0.9817	0.9879	0.9905

**Table 9:** Performance comparison with other methods.

Paper	Best Algorithm	F1-Score		
		Task 1	Task 2	Task 3
Singh et al. [6]	RF	1.0000	1.0000	X
Singh et al. [11]	Ensemble ML	0.997	0.9970	X
MontazeriShatoori et al. [12]	LSTM-based	0.9980	0.999	X
Casanova et al. [24]	BiLSTM	0.9870	0.9990	X
Zebin et al. [16]	Balanced Stacked RF	0.9990	0.9990	X
Casanova et al. [14]	BiLSTM	0.9950	0.9900	X
Stalder [15]	ML	0.9980	0.9890	X
Aggarwal et al. [13]	Ensemble ML	0.9986	0.9999	X
Liu et al. [5]	MFC-DoH (few-shot 20)	X	X	0.9100
MTL-DoHTA	MTL-DoHTA	0.9891	0.9988	0.9837

**Table 10:** Parameter comparison with deep learning methods.

Paper	Model Complexity	Scalability	Number of Parameter (Model)
MontazeriShatoori et al. [12]	Middle	Middle	about 37,000
Casanova et al. [14]	Middle	Middle	72,244
Liu et al. [5]	High	High	1,147,904
MTL-DoHTA	High	High	105,546

## Data Availability

The data presented in this study are available on request from the corresponding author.
